# *N*,*N*′-Bis(4-fluoro­phen­yl)urea

**DOI:** 10.1107/S1600536810016399

**Published:** 2010-05-12

**Authors:** Wan-Sin Loh, Hoong-Kun Fun, S. Sarveswari, V. Vijayakumar, R. Venkat Ragavan

**Affiliations:** a X-ray Crystallography Unit, School of Physics, Universiti Sains Malaysia, 11800 USM, Penang, Malaysia; b Organic Chemistry Division, School of Advanced Sciences, VIT University, Vellore 632 014, India

## Abstract

The asymmetric unit of the title compound, C_13_H_10_F_2_N_2_O, contains one and a half *N*,*N*′-bis­(4-fluoro­phen­yl)urea mol­ecules. One of the mol­ecules has crystallographic twofold rotation symmetry. The benzene rings are twisted from each other by dihedral angles of 29.69 (6)° for the mol­ecule in a general position and 89.83 (6)° for the symmetry-generated mol­ecule. In the crystal structure, a pair of inter­molecular N—H⋯O hydrogen bonds link symmetry-related mol­ecules into chains along the *b* axis, forming *R*_2_^1^(6) ring motifs.

## Related literature

For background to and the biological activity of bis-aryl­ureas, see: Khire *et al.* (2004 ▶); McDonnell *et al.* (2008 ▶); Francisco *et al.* (2004 ▶); Bigi *et al.* (1998 ▶). For the synthetic method, see: Sarveswari & Raja (2006 ▶). For a related structure, see: Jai-nhuknan *et al.* (1997 ▶). For hydrogen-bond motifs, see: Bernstein *et al.* (1995 ▶). For the stability of the temperature controller used for the data collection, see: Cosier & Glazer (1986 ▶).
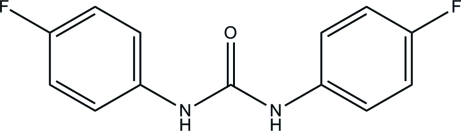


## Experimental

### 

#### Crystal data

C_13_H_10_F_2_N_2_O
*M* *_r_* = 248.23
Monoclinic, 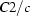

*a* = 67.541 (4) Å
*b* = 4.5750 (3) Å
*c* = 10.7098 (6) Å
β = 95.969 (2)°
*V* = 3291.4 (3) Å^3^
*Z* = 12
Mo *K*α radiation
μ = 0.12 mm^−1^
*T* = 100 K
0.59 × 0.12 × 0.09 mm

#### Data collection

Bruker APEXII DUO CCD area-detector diffractometer
Absorption correction: multi-scan (*SADABS*; Bruker, 2009 ▶) *T*_min_ = 0.932, *T*_max_ = 0.990
21915 measured reflections
5986 independent reflections
4304 reflections with *I* > 2σ(*I*)
*R*_int_ = 0.050

#### Refinement

*R*[*F* ^2^ > 2σ(*F* ^2^)] = 0.048
*wR*(*F* ^2^) = 0.159
*S* = 1.05
5986 reflections
257 parameters
H atoms treated by a mixture of independent and constrained refinement
Δρ_max_ = 0.69 e Å^−3^
Δρ_min_ = −0.51 e Å^−3^

### 

Data collection: *APEX2* (Bruker, 2009 ▶); cell refinement: *SAINT* (Bruker, 2009 ▶); data reduction: *SAINT*; program(s) used to solve structure: *SHELXTL* (Sheldrick, 2008 ▶); program(s) used to refine structure: *SHELXTL*; molecular graphics: *SHELXTL*; software used to prepare material for publication: *SHELXTL* and *PLATON* (Spek, 2009 ▶).

## Supplementary Material

Crystal structure: contains datablocks global, I. DOI: 10.1107/S1600536810016399/lh5040sup1.cif

Structure factors: contains datablocks I. DOI: 10.1107/S1600536810016399/lh5040Isup2.hkl

Additional supplementary materials:  crystallographic information; 3D view; checkCIF report

## Figures and Tables

**Table 1:** Hydrogen-bond geometry (Å, °)

*D*—H⋯*A*	*D*—H	H⋯*A*	*D*⋯*A*	*D*—H⋯*A*
N1*A*—H1*NA*⋯O1*A*^i^	0.83 (2)	2.08 (2)	2.8331 (13)	151.7 (18)
N1*B*—H1*NB*⋯O1*B*^i^	0.89 (3)	2.02 (3)	2.8392 (18)	153.3 (19)
N2*A*—H2*NA*⋯O1*A*^i^	0.855 (18)	2.080 (17)	2.8547 (16)	150.5 (13)

Symmetry code: (i) 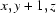.

**Fig. 1.**
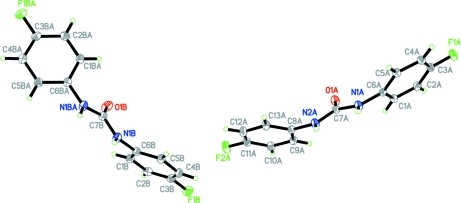
The molecular structure of the title compound, showing 50% probability displacement ellipsoids and the atom-numbering scheme. Atoms with suffix BA [C1BA–C6BA/N1BA/F1BA] are generated by symmetry code -x, y, -z+3/2.

**Fig. 2.**
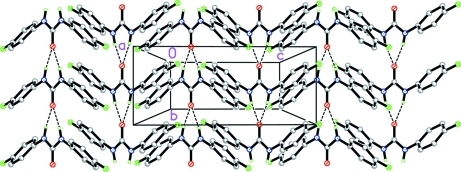
The crystal packing of the title compound, viewed approximately along the a axis, showing R21(6) ring motifs. H atoms not involved in the intermolecular interactions (dashed lines) have been omitted for clarity.

## supplementary crystallographic information

### Comment

The synthesis of bis-arylureas has received considerable attention due to their wide range of biological applications. They act as potential Raf kinase inhibitors (Khire *et al.*, 2004) and antagonists of human vanilloid receptor 1 (VR 1) (McDonnell *et al.*, 2008). Phenyl thiazolylurea derivatives have been reported as inhibitors of Murine receptor A and Murine receptor B (Francisco *et al.*, 2004). Some substituted ureas are used as antidiabetic and tranquilizing drugs, antioxidants in gasoline, corrosion inhibitor and herbicides (Bigi *et al.*, 1998).

The asymmetric unit of the title compound (Fig. 1), comprises of one and a half *N*,*N'*-bis-(4-fluorophenyl)urea molecules. The half molecule has a twofold rotation symmetry, generated by symmetry code -x, y, -z+3/2. In the molecule with suffix A, both benzene rings (C1A–C6A and C8A–C13A) are twisted from each other with a dihedral angle of 29.69 (6)° whereas in molecule with suffix B, the dihedral angle between the benzene rings (C1B–C6B and C1BA–C6BA) is 89.83 (6)°. The structure is comparable to the related structure (Jai-nhuknan *et al.*, 1997).

In the crystal packing (Fig. 2), intermolecular N1A—H1NA···O1A and N2A—H2NA···O1A hydrogen bonds (Table 1) link the adjacent molecules into chains along the *b* axis, forming *R*_2_^1^(6) ring motifs (Bernstein *et al.*, 1995).

### Experimental

The compound *N*,*N'*-bis-(4-fluorophenyl)urea was synthesized using the method available in the literature (Sarveswari & Raja, 2006) and the obtained crude product was recrystallized from absolute ethanol. *M.P.*: 519 K. Yield: 56%.

### Refinement

H1NA, H1NB and H2NA were located from a difference Fourier map and refined freely [N–H = 0.83 (2) to 0.88 (3) Å]. The remaining H atoms were positioned geometrically [C–H = 0.93 Å] and were refined using a riding model, with *U*_iso_(H) = 1.2 *U*_eq_(C). In the final difference Fourier map, the highest peak is 0.20 Å from atom O1B and the deepest hole is 0.45 Å from atom C7B.

### Crystal data

**Table:**

C_13_H_10_F_2_N_2_O	*F*(000) = 1536
*M**_r_* = 248.23	*D*_x_ = 1.503 Mg m^−^^3^
Monoclinic, *C*2/*c*	Mo *K*α radiation, λ = 0.71073 Å
Hall symbol: -C 2yc	Cell parameters from 3635 reflections
*a* = 67.541 (4) Å	θ = 2.4–32.1°
*b* = 4.5750 (3) Å	µ = 0.12 mm^−^^1^
*c* = 10.7098 (6) Å	*T* = 100 K
β = 95.969 (2)°	Block, brown
*V* = 3291.4 (3) Å^3^	0.59 × 0.12 × 0.09 mm
*Z* = 12	

### Data collection

**Table:**

Bruker APEXII DUO CCD area-detector diffractometer	5986 independent reflections
Radiation source: fine-focus sealed tube	4304 reflections with *I* > 2σ(*I*)
graphite	*R*_int_ = 0.050
φ and ω scans	θ_max_ = 32.7°, θ_min_ = 1.8°
Absorption correction: multi-scan (*SADABS*; Bruker, 2009)	*h* = −102→102
*T*_min_ = 0.932, *T*_max_ = 0.990	*k* = −6→6
21915 measured reflections	*l* = −16→16

### Refinement

**Table:**

Refinement on *F*^2^	Primary atom site location: structure-invariant direct methods
Least-squares matrix: full	Secondary atom site location: difference Fourier map
*R*[*F*^2^ > 2σ(*F*^2^)] = 0.048	Hydrogen site location: inferred from neighbouring sites
*wR*(*F*^2^) = 0.159	H atoms treated by a mixture of independent and constrained refinement
*S* = 1.05	*w* = 1/[σ^2^(*F*_o_^2^) + (0.0899*P*)^2^ + 0.1948*P*] where *P* = (*F*_o_^2^ + 2*F*_c_^2^)/3
5986 reflections	(Δ/σ)_max_ < 0.001
257 parameters	Δρ_max_ = 0.69 e Å^−^^3^
0 restraints	Δρ_min_ = −0.51 e Å^−^^3^

### Special details

**Table:**

Experimental. The crystal was placed in the cold stream of an Oxford Cryosystems Cobra open-flow nitrogen cryostat (Cosier & Glazer, 1986) operating at 100.0 (1) K.
Geometry. All esds (except the esd in the dihedral angle between two l.s. planes) are estimated using the full covariance matrix. The cell esds are taken into account individually in the estimation of esds in distances, angles and torsion angles; correlations between esds in cell parameters are only used when they are defined by crystal symmetry. An approximate (isotropic) treatment of cell esds is used for estimating esds involving l.s. planes.
Refinement. Refinement of *F*^2^ against ALL reflections. The weighted *R*-factor wR and goodness of fit *S* are based on *F*^2^, conventional *R*-factors *R* are based on *F*, with *F* set to zero for negative *F*^2^. The threshold expression of *F*^2^ > σ(*F*^2^) is used only for calculating *R*-factors(gt) etc. and is not relevant to the choice of reflections for refinement. *R*-factors based on *F*^2^ are statistically about twice as large as those based on *F*, and *R*- factors based on ALL data will be even larger.

### Fractional atomic coordinates and isotropic or equivalent isotropic displacement parameters (Å^2^)

**Table:**

	*x*	*y*	*z*	*U*_iso_*/*U*_eq_	
F1A	0.244179 (12)	0.5229 (2)	0.33663 (8)	0.02522 (19)	
O1A	0.167883 (13)	0.5447 (2)	0.62122 (9)	0.0198 (2)	
N1A	0.180685 (15)	0.9777 (2)	0.55991 (10)	0.0162 (2)	
N2A	0.153498 (15)	0.9743 (3)	0.67129 (11)	0.0175 (2)	
C1A	0.209007 (17)	0.6417 (3)	0.56407 (11)	0.0163 (2)	
H1AA	0.2064	0.5746	0.6426	0.020*	
C2A	0.225076 (17)	0.5295 (3)	0.50863 (12)	0.0180 (2)	
H2AA	0.2332	0.3860	0.5486	0.022*	
C3A	0.228714 (17)	0.6362 (3)	0.39263 (12)	0.0178 (2)	
C4A	0.217243 (18)	0.8517 (3)	0.33077 (11)	0.0184 (2)	
H4AA	0.2203	0.9236	0.2539	0.022*	
C5A	0.200924 (18)	0.9591 (3)	0.38612 (11)	0.0172 (2)	
H5AA	0.1928	1.1008	0.3451	0.021*	
C6A	0.196784 (16)	0.8543 (3)	0.50280 (10)	0.0142 (2)	
C7A	0.167422 (16)	0.8158 (3)	0.61783 (11)	0.0150 (2)	
C8A	0.138631 (16)	0.8455 (3)	0.73842 (11)	0.0154 (2)	
C9A	0.143599 (18)	0.6409 (3)	0.83227 (12)	0.0187 (2)	
H9AA	0.1568	0.5838	0.8508	0.022*	
C10A	0.128905 (18)	0.5208 (3)	0.89873 (12)	0.0207 (3)	
H10A	0.1320	0.3818	0.9610	0.025*	
C11A	0.109514 (19)	0.6143 (3)	0.86946 (12)	0.0209 (3)	
C12A	0.104185 (18)	0.8202 (3)	0.77910 (12)	0.0219 (3)	
H12A	0.0910	0.8809	0.7630	0.026*	
C13A	0.118945 (18)	0.9362 (3)	0.71211 (12)	0.0193 (2)	
H13A	0.1157	1.0745	0.6497	0.023*	
F2A	0.095139 (12)	0.4948 (2)	0.93363 (9)	0.0309 (2)	
F1B	0.072613 (12)	0.6424 (2)	1.16351 (8)	0.0289 (2)	
O1B	0.0000	0.6661 (3)	0.7500	0.0262 (3)	
N1B	0.013863 (16)	1.0982 (3)	0.82126 (11)	0.0193 (2)	
C1B	0.024299 (18)	0.7613 (3)	0.99248 (12)	0.0200 (2)	
H1BA	0.0112	0.6961	0.9919	0.024*	
C2B	0.03907 (2)	0.6455 (3)	1.07851 (12)	0.0213 (3)	
H2BA	0.0362	0.4998	1.1343	0.026*	
C3B	0.058161 (18)	0.7529 (3)	1.07883 (12)	0.0210 (3)	
C4B	0.063141 (18)	0.9679 (3)	0.99790 (12)	0.0212 (3)	
H4BA	0.0761	1.0385	1.0018	0.025*	
C5B	0.048369 (18)	1.0776 (3)	0.91008 (12)	0.0194 (2)	
H5BA	0.0515	1.2200	0.8533	0.023*	
C6B	0.028914 (17)	0.9741 (3)	0.90722 (12)	0.0170 (2)	
C7B	0.0000	0.9380 (4)	0.7500	0.0181 (3)	
H1NA	0.1792 (3)	1.157 (5)	0.5567 (17)	0.028 (5)*	
H1NB	0.0136 (3)	1.289 (6)	0.8078 (19)	0.043 (6)*	
H2NA	0.1535 (2)	1.160 (4)	0.6624 (16)	0.022 (4)*	

### Atomic displacement parameters (Å^2^)

**Table:**

	*U*^11^	*U*^22^	*U*^33^	*U*^12^	*U*^13^	*U*^23^
F1A	0.0210 (3)	0.0251 (5)	0.0312 (4)	0.0051 (3)	0.0105 (3)	−0.0019 (4)
O1A	0.0215 (4)	0.0090 (4)	0.0302 (5)	−0.0003 (3)	0.0084 (3)	−0.0003 (4)
N1A	0.0180 (4)	0.0082 (5)	0.0231 (5)	0.0010 (4)	0.0059 (4)	0.0014 (4)
N2A	0.0184 (4)	0.0094 (5)	0.0258 (5)	0.0014 (4)	0.0076 (4)	0.0010 (4)
C1A	0.0170 (5)	0.0144 (6)	0.0175 (5)	0.0004 (4)	0.0018 (4)	0.0011 (4)
C2A	0.0159 (5)	0.0147 (6)	0.0232 (6)	0.0025 (4)	0.0015 (4)	0.0012 (5)
C3A	0.0148 (5)	0.0171 (6)	0.0220 (5)	0.0005 (4)	0.0040 (4)	−0.0041 (5)
C4A	0.0195 (5)	0.0187 (6)	0.0174 (5)	−0.0004 (5)	0.0046 (4)	0.0001 (5)
C5A	0.0183 (5)	0.0151 (6)	0.0181 (5)	0.0007 (5)	0.0021 (4)	0.0006 (4)
C6A	0.0150 (4)	0.0106 (5)	0.0170 (5)	−0.0005 (4)	0.0017 (4)	−0.0015 (4)
C7A	0.0153 (4)	0.0119 (5)	0.0178 (5)	0.0006 (4)	0.0015 (4)	−0.0001 (4)
C8A	0.0157 (4)	0.0114 (5)	0.0195 (5)	−0.0011 (4)	0.0039 (4)	−0.0013 (4)
C9A	0.0178 (5)	0.0176 (6)	0.0205 (5)	−0.0003 (5)	0.0016 (4)	0.0011 (5)
C10A	0.0212 (5)	0.0207 (7)	0.0205 (6)	0.0006 (5)	0.0039 (4)	0.0035 (5)
C11A	0.0201 (5)	0.0200 (6)	0.0239 (6)	−0.0038 (5)	0.0078 (4)	−0.0015 (5)
C12A	0.0161 (5)	0.0215 (7)	0.0286 (6)	0.0010 (5)	0.0044 (4)	0.0005 (5)
C13A	0.0174 (5)	0.0168 (6)	0.0238 (6)	0.0019 (5)	0.0030 (4)	0.0020 (5)
F2A	0.0247 (4)	0.0319 (5)	0.0384 (5)	−0.0037 (4)	0.0150 (3)	0.0060 (4)
F1B	0.0284 (4)	0.0301 (5)	0.0263 (4)	0.0062 (4)	−0.0060 (3)	0.0031 (4)
O1B	0.0302 (7)	0.0107 (6)	0.0351 (7)	0.000	−0.0093 (6)	0.000
N1B	0.0177 (4)	0.0112 (5)	0.0280 (5)	−0.0008 (4)	−0.0017 (4)	0.0004 (4)
C1B	0.0194 (5)	0.0172 (6)	0.0235 (6)	−0.0020 (5)	0.0031 (4)	−0.0001 (5)
C2B	0.0275 (6)	0.0165 (6)	0.0200 (5)	−0.0009 (5)	0.0029 (4)	0.0015 (5)
C3B	0.0216 (5)	0.0203 (6)	0.0201 (5)	0.0039 (5)	−0.0024 (4)	−0.0008 (5)
C4B	0.0168 (5)	0.0223 (7)	0.0244 (6)	−0.0004 (5)	0.0011 (4)	−0.0012 (5)
C5B	0.0184 (5)	0.0180 (6)	0.0218 (5)	−0.0010 (5)	0.0022 (4)	0.0007 (5)
C6B	0.0171 (5)	0.0123 (6)	0.0213 (5)	0.0007 (4)	0.0010 (4)	−0.0016 (4)
C7B	0.0170 (7)	0.0136 (8)	0.0234 (8)	0.000	0.0005 (6)	0.000

### Geometric parameters (Å, °)

**Table:**

F1A—C3A	1.3601 (13)	C10A—H10A	0.9300
O1A—C7A	1.2412 (15)	C11A—F2A	1.3611 (14)
N1A—C7A	1.3606 (15)	C11A—C12A	1.371 (2)
N1A—C6A	1.4190 (15)	C12A—C13A	1.3925 (17)
N1A—H1NA	0.83 (2)	C12A—H12A	0.9300
N2A—C7A	1.3602 (15)	C13A—H13A	0.9300
N2A—C8A	1.4223 (15)	F1B—C3B	1.3589 (15)
N2A—H2NA	0.85 (2)	O1B—C7B	1.244 (2)
C1A—C2A	1.3886 (16)	N1B—C7B	1.3593 (15)
C1A—C6A	1.3948 (17)	N1B—C6B	1.4173 (16)
C1A—H1AA	0.9300	N1B—H1NB	0.88 (3)
C2A—C3A	1.3805 (18)	C1B—C2B	1.3902 (18)
C2A—H2AA	0.9300	C1B—C6B	1.3921 (18)
C3A—C4A	1.3799 (18)	C1B—H1BA	0.9300
C4A—C5A	1.3941 (16)	C2B—C3B	1.3798 (19)
C4A—H4AA	0.9300	C2B—H2BA	0.9300
C5A—C6A	1.3937 (16)	C3B—C4B	1.376 (2)
C5A—H5AA	0.9300	C4B—C5B	1.3916 (18)
C8A—C9A	1.3888 (18)	C4B—H4BA	0.9300
C8A—C13A	1.3935 (16)	C5B—C6B	1.3942 (17)
C9A—C10A	1.3929 (17)	C5B—H5BA	0.9300
C9A—H9AA	0.9300	C7B—N1B^i^	1.3593 (15)
C10A—C11A	1.3824 (18)		
			
C7A—N1A—C6A	123.39 (11)	C9A—C10A—H10A	121.0
C7A—N1A—H1NA	118.3 (13)	F2A—C11A—C12A	118.92 (12)
C6A—N1A—H1NA	118.3 (13)	F2A—C11A—C10A	118.03 (12)
C7A—N2A—C8A	123.17 (11)	C12A—C11A—C10A	123.05 (12)
C7A—N2A—H2NA	118.4 (11)	C11A—C12A—C13A	118.42 (12)
C8A—N2A—H2NA	118.4 (11)	C11A—C12A—H12A	120.8
C2A—C1A—C6A	120.49 (11)	C13A—C12A—H12A	120.8
C2A—C1A—H1AA	119.8	C12A—C13A—C8A	120.09 (12)
C6A—C1A—H1AA	119.8	C12A—C13A—H13A	120.0
C3A—C2A—C1A	118.30 (11)	C8A—C13A—H13A	120.0
C3A—C2A—H2AA	120.8	C7B—N1B—C6B	123.66 (12)
C1A—C2A—H2AA	120.8	C7B—N1B—H1NB	116.0 (14)
F1A—C3A—C4A	118.63 (11)	C6B—N1B—H1NB	120.2 (14)
F1A—C3A—C2A	118.60 (11)	C2B—C1B—C6B	120.46 (11)
C4A—C3A—C2A	122.77 (11)	C2B—C1B—H1BA	119.8
C3A—C4A—C5A	118.48 (11)	C6B—C1B—H1BA	119.8
C3A—C4A—H4AA	120.8	C3B—C2B—C1B	118.17 (13)
C5A—C4A—H4AA	120.8	C3B—C2B—H2BA	120.9
C6A—C5A—C4A	120.08 (12)	C1B—C2B—H2BA	120.9
C6A—C5A—H5AA	120.0	F1B—C3B—C4B	118.73 (12)
C4A—C5A—H5AA	120.0	F1B—C3B—C2B	118.40 (13)
C5A—C6A—C1A	119.84 (11)	C4B—C3B—C2B	122.87 (12)
C5A—C6A—N1A	118.94 (11)	C3B—C4B—C5B	118.59 (12)
C1A—C6A—N1A	121.14 (10)	C3B—C4B—H4BA	120.7
O1A—C7A—N2A	122.47 (11)	C5B—C4B—H4BA	120.7
O1A—C7A—N1A	122.79 (11)	C4B—C5B—C6B	120.01 (13)
N2A—C7A—N1A	114.74 (11)	C4B—C5B—H5BA	120.0
C9A—C8A—C13A	120.06 (11)	C6B—C5B—H5BA	120.0
C9A—C8A—N2A	121.00 (10)	C1B—C6B—C5B	119.87 (12)
C13A—C8A—N2A	118.90 (11)	C1B—C6B—N1B	120.79 (11)
C8A—C9A—C10A	120.28 (11)	C5B—C6B—N1B	119.26 (12)
C8A—C9A—H9AA	119.9	O1B—C7B—N1B^i^	122.63 (9)
C10A—C9A—H9AA	119.9	O1B—C7B—N1B	122.63 (8)
C11A—C10A—C9A	118.08 (12)	N1B^i^—C7B—N1B	114.74 (17)
C11A—C10A—H10A	121.0		

Symmetry codes: (i) −*x*, *y*, −*z*+3/2.

### Hydrogen-bond geometry (Å, °)

**Table:**

*D*—H···*A*	*D*—H	H···*A*	*D*···*A*	*D*—H···*A*
N1A—H1NA···O1A^ii^	0.83 (2)	2.08 (2)	2.8331 (13)	151.7 (18)
N1B—H1NB···O1B^ii^	0.89 (3)	2.02 (3)	2.8392 (18)	153.3 (19)
N2A—H2NA···O1A^ii^	0.855 (18)	2.080 (17)	2.8547 (16)	150.5 (13)

Symmetry codes: (ii) *x*, *y*+1, *z*.
